# Effectiveness of a strategy that uses educational games to implement clinical practice guidelines among Spanish residents of family and community medicine (e-EDUCAGUIA project): a clinical trial by clusters

**DOI:** 10.1186/s13012-016-0425-3

**Published:** 2016-05-17

**Authors:** Isabel del Cura-González, Juan A. López-Rodríguez, Teresa Sanz-Cuesta, Ricardo Rodríguez-Barrientos, Jesús Martín-Fernández, Gloria Ariza-Cardiel, Elena Polentinos-Castro, Begoña Román-Crespo, Esperanza Escortell-Mayor, Milagros Rico-Blázquez, Virginia Hernández-Santiago, Amaya Azcoaga-Lorenzo, Elena Ojeda-Ruiz, Ana I González-González, José F Ávila-Tomas, Jaime Barrio-Cortés, José M Molero-García, Raul Ferrer-Peña, María Eugenia Tello-Bernabé, Mar Trujillo-Martín, AND Educaguia Group

**Affiliations:** 1 Unidad de Apoyo a la Investigación, Gerencia Asistencial de Atención Primaria, Servicio Madrileño de Salud, Calle San Martín de Porres, 6, 28035 Madrid, Spain; 2 Área Medicina Preventiva y Salud Pública, Facultad de Ciencias de la Salud, Universidad Rey Juan Carlos, Madrid, Spain; 3 Departamento Medicina, Facultad de Ciencias de la Salud, Universidad Rey Juan Carlos, Madrid, Spain; 4 Red de Investigación en Servicios de Salud en Enfermedades Crónicas (REDISSEC), Madrid, Spain; 5 Grupo Infecciosas Sociedad Madrileña de Medicina Familiar y Comunitaria, (SoMaMFyC), Madrid, Spain; 6 C.S. Villamanta, Dirección Asistencial Oeste, Gerencia Asistencial de Atención Primaria, Servicio Madrileño de Salud, Servicio Madrileño de Salud, Calle San Martín de Porres, 6, 28035 Madrid, Spain; 7 Unidad Docente Multiprofesional Atención Familiar y Comunitaria Oeste, Unidad de Apoyo a la Investigación, Gerencia Asistencial de Atención Primaria, Servicio Madrileño de Salud, Calle San Martín de Porres, 6, 28035 Madrid, Spain; 8 Unidad Docente Multiprofesional Atención Familiar y Comunitaria Norte, Unidad de Apoyo a la Investigación, Gerencia Asistencial de Atención Primaria, Servicio Madrileño de Salud, Calle San Martín de Porres, 6, 28035 Madrid, Spain; 9 Mackenzie Building. Kirsty Semple Way, Ninewells Hospital & Medical School, Dundee, DD2 4PF UK; 10 C.S. Pintores. Gerencia Asistencial de Atención Primaria, Servicio Madrileño de Salud, Pintor Rosales, S/N, 28982 Parla, Madrid Spain; 11 Servicio Medicina Preventiva, Hospital Universitario Severo Ochoa, Avenida de Orellana, s/n, 28911 Leganés, Madrid Spain; 12 Grupo Medicina Basada en la Evidencia de la Sociedad Española de Medicina Familiar y Comunitaria, (SoMaMFyC), Madrid, Spain; 13 C.S. Santa Isabel. Gerencia Asistencial de Atención Primaria. Servicio Madrileño de Salud, Paseo de Colón, 3, 28911 Leganés, Madrid Spain; 14 Grupo Nuevas Tecnologías Sociedad Madrileña de Medicina Familiar Y Comunitaria, (SOMAMFYC), Madrid, Spain; 15 C.S. Jazmín, Unidad Docente Multiprofesional Centro, Gerencia Asistencial de Atención Primaria, Servicio Madrileño de Salud, Madrid, Spain; 16 C.S. San Andrés, Calle Alberto Palacios 22, 28021 Madrid, Spain; 17 C.S. Entrevias. Gerencia Asistencial de Atención Primaria. Servicio Madrileño de Salud, C/Pedroches c/v Campiña, 28053 Madrid, Spain; 18 Departamento de fisioterapia, Centro Superior de Estudios Universitarios La Salle, Universidad Autónoma de Madrid, Madrid, Spain; 19 C.S. El Naranjo, Calle de Avilés, 2, 28942 Fuenlabrada, Madrid Spain; 20 Servicio de Evaluación y Planificación, Dirección del Servicio Canario de la Salud, Fundación Canaria de Investigación Sanitaria (FUNCANIS), Camino Candelaria, 44. C.S. San Isidro-El Chorrillo, 38109 El Rosario, Tenerife España

**Keywords:** Health personnel/education, Professional competence, Games, Experimental, Problem solving, Practice guidelines as a topic

## Abstract

**Background:**

Clinical practice guidelines (CPGs) have been developed with the aim of helping health professionals, patients, and caregivers make decisions about their health care, using the best available evidence. In many cases, incorporation of these recommendations into clinical practice also implies a need for changes in routine clinical practice. Using educational games as a strategy for implementing recommendations among health professionals has been demonstrated to be effective in some studies; however, evidence is still scarce. The primary objective of this study is to assess the effectiveness of a teaching strategy for the implementation of CPGs using educational games (e-learning EDUCAGUIA) to improve knowledge and skills related to clinical decision-making by residents in family medicine. The primary objective will be evaluated at 1 and 6 months after the intervention. The secondary objectives are to identify barriers and facilitators for the use of guidelines by residents of family medicine and to describe the educational strategies used by Spanish teaching units of family and community medicine to encourage implementation of CPGs.

**Methods/design:**

We propose a multicenter clinical trial with randomized allocation by clusters of family and community medicine teaching units in Spain. The sample size will be 394 residents (197 in each group), with the teaching units as the randomization unit and the residents comprising the analysis unit. For the intervention, both groups will receive an initial 1-h session on clinical practice guideline use and the usual dissemination strategy by e-mail. The intervention group (e-learning EDUCAGUIA) strategy will consist of educational games with hypothetical clinical scenarios in a virtual environment.

The primary outcome will be the score obtained by the residents on evaluation questionnaires for each clinical practice guideline. Other included variables will be the sociodemographic and training variables of the residents and the teaching unit characteristics. The statistical analysis will consist of a descriptive analysis of variables and a baseline comparison of both groups. For the primary outcome analysis, an average score comparison of hypothetical scenario questionnaires between the EDUCAGUIA intervention group and the control group will be performed at 1 and 6 months post-intervention, using 95 % confidence intervals. A linear multilevel regression will be used to adjust the model.

**Discussion:**

The identification of effective teaching strategies will facilitate the incorporation of available knowledge into clinical practice that could eventually improve patient outcomes. The inclusion of information technologies as teaching tools permits greater learning autonomy and allows deeper instructor participation in the monitoring and supervision of residents. The long-term impact of this strategy is unknown; however, because it is aimed at professionals undergoing training and it addresses prevalent health problems, a small effect can be of great relevance.

**Trial registration:**

ClinicalTrials.gov: NCT02210442.

## Background

Clinical practice guidelines (CPGs) are a set of “systematically developed statements to assist practitioner and patient decisions about appropriate health care for specific clinical circumstances” [1]. CPGs are developed with the aim of helping health professionals, patients, and caregivers make decisions about their health care, using the best available evidence. Several studies have proven that following the recommendations of certain CPGs improves patients’ health outcomes [2, 3]. In Spain, the National Health System Quality Plan of the Spanish Ministry of Health and Social Policy (GuiaSalud) has developed high-quality CPGs and made them available to all health professionals; these guidelines demonstrate great teaching potential that could improve health care. Many of these CPGs specifically target primary care health professionals [4].

Greater publication and dissemination of a CPG does not necessarily result in its systematic use. In many cases, incorporation of the CPG recommendations into clinical practice also implies a need for changes in routine clinical practice [5, 6]; thus, a subsequent implementation stage must be planned. Implementing a CPG involves launching a process aimed at the successful understanding and application of set recommendations through the use of effective communication strategies to promote change. This process constitutes one of the basic phases of creating a CPG and requires a planning stage, in which special attention must be given to the context—both institutional and social—and the identification of barriers and facilitators that will determine later use of the CPG [5]. Several studies [7–9] have assessed factors that could influence implementation of evidence-based CPGs as well as selection of the best implementation strategies. CPGs are considered excellent teaching tools for acquiring skills and assessing clinical practice [10–12]. They provide information on the usefulness of diagnostic tests and their application in clinical practice. The important role played by CPGs in enabling resident doctors to prescribe appropriately and to identify potential conflicts of interest must be highlighted [13]. The educational program for family and community medicine was the first in Spain to incorporate CPGs as tools to enable the acquisition of essential competencies. In this program, understanding the conceptual basis of clinical practice is considered the first priority (i.e., an essential competence must be acquired by all residents, whose absence would result in doubt about the resident’s aptitude) [14], along with knowledge of evidence-based medicine (EBM) as a tool for clinical management through the use of CPGs, focusing on the patient.

There is evidence of limited CPG use by both residents and their mentors [15]. Several studies have confirmed the limited use of CPGs by resident doctors in various areas, such as the prescription of antibiotics, preventive health activities, or handling prevalent pathologies such as hypertension or diabetes. A study evaluating antibiotic prescription in the treatment of urinary tract infections by family medicine residents at the Mayo Clinic found that only 30 % of prescriptions had been prescribed in accordance with the recommendations provided in the corresponding CPG [16]. Another study used a sample of 376 residents and explored how to approach renal failure treatment using a CPG that contained hypothetical clinical scenarios. The authors reported a significant lack of knowledge of the relevant risk factors, with the exception of hypertension [17]. In addition, the majority of the 127 residents included in a study assessing subjects’ knowledge regarding screening for colon cancer reported family history of colorectal cancer as a screening factor; however, their knowledge regarding screening recommendations in cases of familial colon polyposis was limited [18]. The effectiveness of the different strategies aimed at increasing adherence to CPG recommendations has previously been evaluated, and most have been shown to improve adherence; the implementation of educational programs, mostly as part of more complex interventions, has also been shown to improve outcomes [19–21]. Some of these strategies specifically targeted resident doctors [17].

The teaching potential of educational games in stimulating learning and individuals’ ability to analyze, synthesize, and evaluate information has been widely studied [22]. In previous years, we have witnessed the incorporation of games as virtual educational tools for supporting the training of students and resident doctors [23, 24]. The inclusion of educational games as a strategy for implementing recommendations among health professionals and resident doctors counts on broad acceptance and helps students acquire new knowledge and abilities, in addition to improving attitudes in an interactive, playful way, thus improving adherence to recommendations [25, 26]. A Cochrane collaboration review concluded that there was no evidence supporting the effectiveness of educational games in the training of health professionals, and given their potential, it seems necessary to perform quality research on the impact of these games on health professional training. Research must include the evaluation of results regarding professional performance, decision-making, the clinical care process, and, if possible, patient outcomes [27, 28].

Our proposal regarding educational games [29] focuses on the guidelines approved by the National Health System Quality Plan of the Spanish Ministry of Health and Social Policy. The selection criteria of guidelines include their applicability to severe or chronic, highly prevalent pathologies in primary health care that are included in the relevant areas within which residents must acquire competencies. Several members of this trial’s research team have been part of the working groups that developed these guidelines and have participated in their subsequent dissemination and implementation [30].

Teaching strategies that improve resident doctors’ application of the CPG recommendations could have a direct impact on the improvement of health care. This impact would be particularly significant in the case of family medicine residents because more than 40 % of doctors who start their residency in Spain will train as family medicine doctors. These professionals will address the health problems of more than 90 % of the population; hence, any effective intervention in this group could have a significant effect.

### Hypothesis

A teaching strategy based on the incorporation of educational games for the implementation of CPGs, compared with the usual dissemination strategy, will improve knowledge and abilities related to clinical decision-making by residents in family medicine. A difference of at least 10 points between the EDUCAGUIA intervention group and the control group in the evaluation questionnaires will be considered statistically significant.

### Objectives

#### Primary

The primary objective of this study is to assess the effectiveness of a teaching strategy for the implementation of CPGs using educational games (e-learning EDUCAGUIA) to improve knowledge and abilities related to clinical decision-making by residents in family medicine, in contrast with typical CPG dissemination. The primary outcome will be evaluated at 1 and 6 months after the intervention.

#### Secondary

1. To describe the adherence to educational games and their acceptance by resident doctors
2. To describe the educational strategies used by Spanish family and community medicine teaching units to encourage CPG implementation
3. To identify barriers and facilitators for the use of CPGs by residents in family medicine

## Methods/design

### Design

A multicenter clinical trial with randomized allocation by clusters.

Phase I will consist of a survey collected from family medicine residency program directors in Spain, the development of the educational games interface, and the elaboration of the competence tests.

**Fig. 1:**
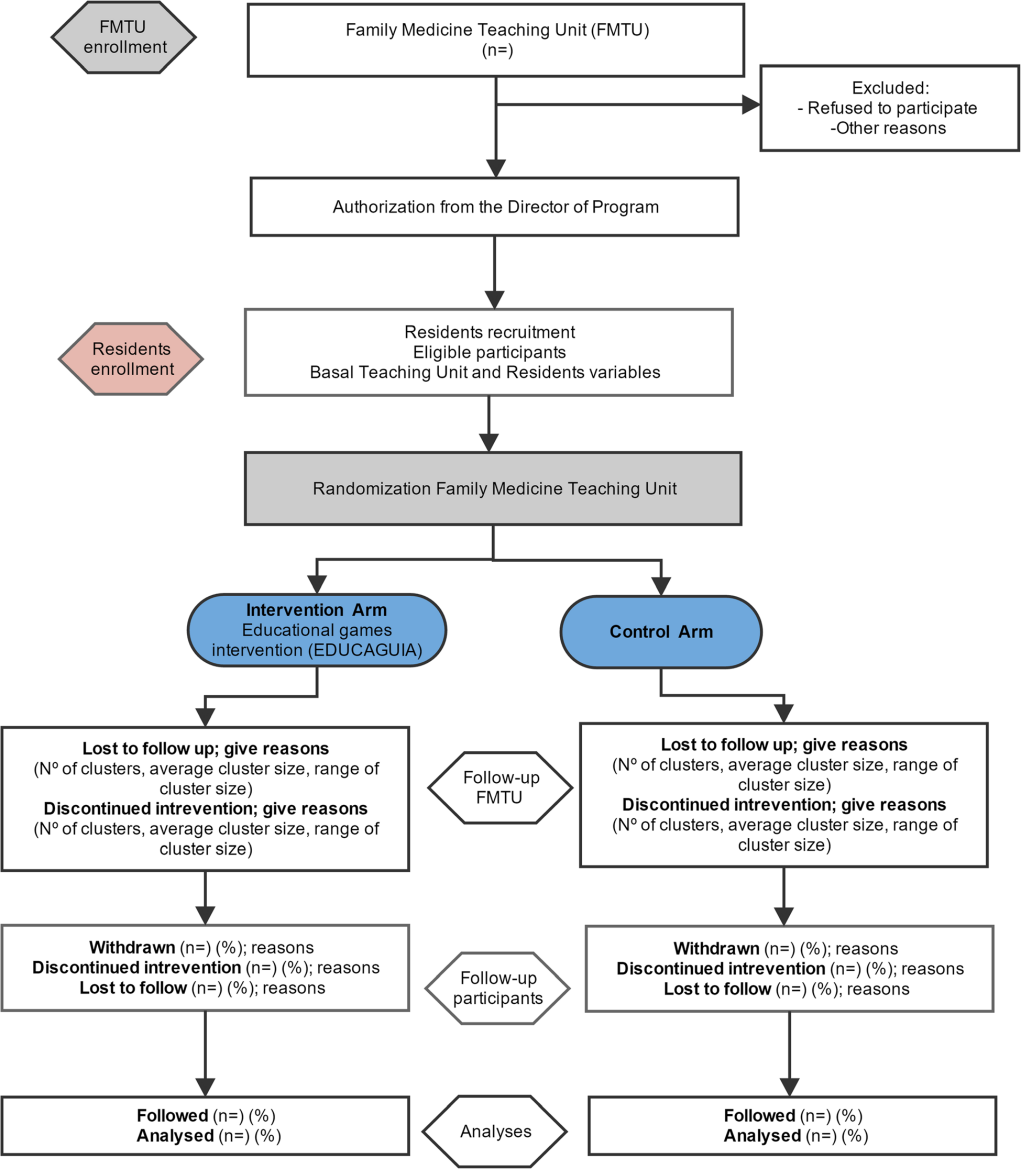
Flow diagram

Phase II will comprise a qualitative study to identify barriers and facilitators to the guideline implementation (Fig. 1).

### Setting

Teaching units of family and community medicine (TUFCM) in Spain.

### Study population

1. Teaching units of family and community medicine inclusion criteria
  - Legal criteria: having Spanish Ministry of Health approval for family medicine teaching to resident doctors and nurses
  - Residency program director agreement for trial participation
2. Family medicine residents’ inclusion criteria
  c. Third- and fourth-year residents belonging to teaching units of family and community medicine
  d. Not opposed to participation

### Recruitment

All Spanish teaching units will be contacted by mail offering participation via an agreement form to participate in the EDUCAGUIA study. TUFCM agreeing to participate to the study will be randomly assigned to one of the two groups: the intervention or the control group.

### Sample size

The sample size has been calculated considering a relevant post-intervention average difference of 10 points on a 100-point evaluation questionnaire between groups and a standard deviation of 0.5 [31]. Assuming an alpha error of 0.05 and a beta error of 0.10, 84 subjects are required for each group. Because randomization is performed by clusters, the sample size has been adjusted to take into account the design effect. Thus, for an average of 30 residents per teaching unit, and an intraclass correlation coefficient (ICC) of 0.03 (literature shows an ICC of 0–0.052 for similar teaching interventions) [32], a design effect of 1.87 has been obtained. With these assumptions, and expecting a 25 % loss to follow-up after a 6-month period, the required sample size is 394 (197 per group).

### Randomization

Allocation will be performed by clusters, and the randomizing unit will be the teaching units. The analysis unit will be the residents. Randomization will be performed by using the Epidat 4.1 software module to assign patients’ treatments. The intervention “e-learning EDUCAGUIA” will be regarded as the treatment, and the routine CPG dissemination system will be used as a control variable. To obtain the same number of centers in both the intervention and control groups, the option of balanced groups will be chosen.

### Intervention

Both groups will attend an initial 60-min session comprising the following content: CPGs—what they are and where to find them; information on and presentation of the GuiaSalud website and a link to the guidelines library; and instructions for downloading guidelines.

### For the control group

Routine dissemination of clinical guidelines will be sent via e-mail. The link to the CPGs from the GuiaSalud website will be provided.

### For the e-EDUCAGUIA intervention group

The e-learning EDUCAGUIA is a complex intervention that combines several components based on an educational game with a ranking and a competitive combination of game styles. It has been designed by a multidisciplinary team of primary healthcare professionals with experience in training students and residents, elaborating CPGs and developing new technologies as teaching tools. After routine disseminations of clinical guidelines via e-mail, as in the control group, residents belonging to the teaching units allocated in this intervention arm will be invited to access the educational games’ online training demo by e-mail. They will then have access to the e-learning EDUCAGUIA application software for one month.

**Fig. 2:**
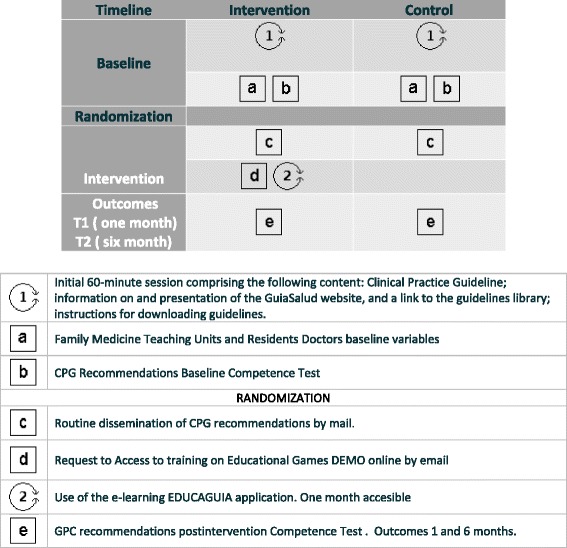
Complex intervention pat plot. e-EDUCAGUIA

Each guideline comprises different clinical scenarios related to several recommendation areas, each encompassing five dimensions (e.g., diagnosis, treatment, referral, information, and patient health education). The application will generate a report for each “game” or training session completed by the residents and will rank them accordingly to enable comparisons with the rest of the guideline users. Figure 2 shows the various components of both the control and the e-EDUCAGUIA intervention groups [33, 34].

### Competence assessment in both groups

An evaluation of baseline competence will be performed for each guideline prior to its presentation, and two further tests will be carried out at 1 and 6 months after the intervention. The test will evaluate the level of knowledge and decision-making in relation to ten hypothetical cases of routine clinical practice that are related to the content of each guideline; the test will yield a maximum score of 100 and will be completed within 45 min.

### Variables

The primary outcome variable will be the participant score obtained in the test assessing competence 1 month after the intervention. The secondary outcome variable will be the competence test score 6 months after the intervention.

- Teaching unit variables
  Sociodemographic characteristics of the program director and technician: sex, age, specialty, academic level, time spent in that position, experience in elaborating guidelines or recommendation, and training in EBM
  Unit characteristics: total number of residents (R1, R2, R3, R4); position of the teaching unit in the ranking, obtaining residency positions in the last three contests; a teaching plan that includes training on EBM/CPG in the unit; and the number of EBM/CPG training hours in the past year
- Resident doctor variables
  Sociodemographic: age, sex, nationality, residency year
  Position in the national exams to access specialty (MIR number); grades obtained in R-1 and R-2 residency years (apt, outstanding, excellent); completion of other specialties; year of obtaining a university degree in medicine; and PhD (yes/no)
  Training: specific training on EBM in the past 5 years and corresponding hours of training, training on critical reading capabilities and corresponding hours of training, having attended formative sessions on how to use CPGs, and corresponding hours of training
  Other variables to achieve secondary objectives: adherence to CPGs, measured as time spent using the application.

### All variables will be collected in an electronic case report form

For secondary objective 3, each teaching unit and mentor will receive an e-mail containing information on the project and inviting them to complete the survey with the link included. The questionnaire has been developed by the research team.

For secondary objective 4, a study using a qualitative methodology was performed in a phase prior to the beginning of the trial, including discussion groups that studied the barriers and facilitators in the use of CPGs. Using the results from that study, along with the questionnaire developed by Sola et al. [35] for Spanish doctors, a new questionnaire will be created that will be sent to the family medicine residents by the teaching units.

### Statistical analysis

1. Descriptive and exploratory analysis: This analysis will include participants’ baseline characteristics and outcomes, means and standard deviations (SD) for continuous variables, and proportions for categorical variables with its corresponding 95 % confidence interval (CI). Baseline comparison between the two groups in terms of the score competence test and descriptive and prognostic factors will be performed by bi-variant statistical tests.
2. Inferential analysis: Student’s *t* test with a 95 % CI will be performed to compare both groups’ results in the competence test in terms of score at baseline and 1- and 6-month post-implementation of each CPG recommendation.
3. Multilevel analysis: a mixed model will be constructed for the evaluation of the influence of the teaching unit on the effectiveness of the intervention. The dependent variable will be the score obtained in the competence test. The model will consist of two levels: resident doctors will comprise the first level and the teaching unit will comprise the second.
4. Dropouts and missing data

Because this trial will use an online system to allocate participants and collect baseline and outcome data, participants can leave the study at any time. To minimize the possibility of dropouts and missing data, several strategies will be considered. If a participant leaves the study before randomization, the participant will be excluded and we will not consider this as missing participant data.

All statistical tests will be performed with an intention-to-treat analysis. The last observation carried forward will be used for missing data imputation, and there will be report analyses with and without imputation [36].

Significance will be set at *p* < .05. Calculations will be performed using MLwiN and STATA v.12 software with specific related modules.

### Ethical and legal aspects

The trial will fulfill the standards of Good Clinical Practice and the principles of the last Helsinki Declaration (Brazil, 2013). The project has been approved by the Clinical Research Ethics Committee of the Alcorcón Foundation University Hospital in Madrid and the Central Committee for Research of the Primary Care Management of the Community of Madrid. In accordance with Spanish Law 15/1999 on personal data protection, confidentiality and anonymity will be guaranteed both at the execution phase of the trial and during the presentations or publications derived from it.

## Discussion

Teaching units from across Spain will participate in this clinical trial, which anticipates high external outcome validity. Additionally, this intervention might provide information to consider for training in other specialties.

The intervention to be assessed cannot be masked, which could influence its effect. Because there is a possibility of contamination bias among residents, randomization by clusters has been proposed, under the assumption that the number of clusters will be sufficient for each group. Geographic dispersion of the teaching units reduces the possibility of contamination.

Teaching strategies are complex interventions; the design of educational games as an interactive, self-learning application allows for standardization of the intervention.

The considerable variability foreseen between teaching units regarding training in CPGs could imply that some residents have better training with some of these CPGs, which could result in a lack of improvement in these residents’ decision-making scores (competency test score). However, this possibility will be adjusted using the training variables of both the teaching units and the residents.

To evaluate the intervention’s effectiveness, the primary measurement will take place 1 month after implementation; at this time, our primary objective will be to confirm the effectiveness of the e-learning EDUCAGUIA strategy as a learning tool. Its effect at 6 months after the intervention might not remain, which would lead to a study on the effectiveness of reinforcement interventions. Measuring the primary response at 6 months was considered an option, but the characteristics of training during residency periods make it possible for the residents to acquire the knowledge and abilities of the intervention during rotations or other courses within that time period, thus hindering the isolation of the intervention effect.

Our proposal, which follows the recommendations by the Cochrane review, attempts to overcome the intervention effectiveness measured solely as an increase in knowledge. Therefore, our proposed outcome measure is clinical decision-making in hypothetical scenarios, which is a more realistic approach toward the implementation of guideline recommendations in the decision-making processes of health professionals. Currently, we cannot measure the outcome for all patients being attended by all residents who will receive training for all CPGs. Therefore, our aim is to evaluate at least a representative sample; in the case of recommendations for antibiotic prescription guidelines, we will evaluate the use of computerized clinical records and isolate the decisions of residents and mentors.

The identification of effective teaching strategies will facilitate the incorporation of available knowledge into clinical practice that could eventually improve patient outcomes. The inclusion of information technologies as teaching tools, adapted to training methodologies for adults, permits greater learning autonomy and simultaneously acts as a teaching tool by allowing greater instructor participation in the monitoring and supervision of residents in the relevant knowledge area. The long-term impact of this strategy is unknown; however, because it is aimed at professionals undergoing training (who must acquire the correct habits and abilities) and it addresses prevalent health problems, a small effect can be of great relevance.

## Abbreviations

CI: confidence interval
CPG: clinical practice guideline
EBM: evidence-based medicine
